# Oxidative Stress Biomarkers in Male Infertility: Established Methodologies and Future Perspectives

**DOI:** 10.3390/genes15050539

**Published:** 2024-04-25

**Authors:** Filomena Mottola, Ilaria Palmieri, Maria Carannante, Angela Barretta, Shubhadeep Roychoudhury, Lucia Rocco

**Affiliations:** 1 Department of Environmental, Biological and Pharmaceutical Sciences and Technologies, University of Campania Luigi Vanvitelli, 81100 Caserta, Italy; filomena.mottola@unicampania.it (F.M.); ilaria.palmieri@unicampania.it (I.P.); maria.carannante@unicampania.it (M.C.); angela.barretta@virgilio.it (A.B.); 2 Department of Life Science and Bioinformatics, Assam University, Silchar 788011, India; shubhadeep1@gmail.com

**Keywords:** oxidative stress, sperm DNA damage, epigenetic alterations, omics, infertility

## Abstract

Male fertility can be affected by oxidative stress (OS), which occurs when an imbalance between the production of reactive oxygen species (ROS) and the body’s ability to neutralize them arises. OS can damage cells and influence sperm production. High levels of lipid peroxidation have been linked to reduced sperm motility and decreased fertilization ability. This literature review discusses the most commonly used biomarkers to measure sperm damage caused by ROS, such as the high level of OS in seminal plasma as an indicator of imbalance in antioxidant activity. The investigated biomarkers include 8-hydroxy-2-deoxyguanosine acid (8-OHdG), a marker of DNA damage caused by ROS, and F2 isoprostanoids (8-isoprostanes) produced by lipid peroxidation. Furthermore, this review focuses on recent methodologies including the NGS polymorphisms and differentially expressed gene (DEG) analysis, as well as the epigenetic mechanisms linked to ROS during spermatogenesis along with new methodologies developed to evaluate OS biomarkers. Finally, this review addresses a valuable insight into the mechanisms of male infertility provided by these advances and how they have led to new treatment possibilities. Overall, the use of biomarkers to evaluate OS in male infertility has supplied innovative diagnostic and therapeutic approaches, enhancing our understanding of male infertility mechanisms.

## 1. Introduction

Male fertility can be affected by oxidative stress (OS), a condition in which a redox imbalance occurs between the production of reactive oxygen species (ROS) and the cells’ antioxidant protection systems. In physiological concentrations, ROS are necessary for proper spermatic functions such as the acrosome reaction, capacitation, and sperm–oocyte fusion [1]. However, an excess of ROS leads to OS, causing structural and functional damage to sperm cells, including lipid peroxidation, protein oxidation, and DNA damage, resulting in compromised male fertility and a risk of genetic mutations in offspring [2,3]. Elevated ROS concentrations, found in seminal fluid samples from infertile men, are often due to pathological conditions or environmental factors such as infections, smoking, alcoholism, and diet [4,5,6,7].

Exposure to environmental pollutants can affect sperm parameters and sperm DNA quality, resulting in cell apoptosis [8].

Insecticides, pesticides, and environmental toxins (heavy metals, bisphenol A, and dioxins) lead to a reduction in male fertility, causing an alteration in sperm DNA both directly and indirectly, through the production of ROS, with a consequent reduction in the number of ejaculated spermatozoa and the presence of morphologically abnormal spermatozoa in seminal fluid [8,9,10,11,12]. Associations have also been found between the urinary concentrations of particular oxidative substances and sperm apoptosis [13,14].

Spermatozoa are vulnerable to oxidative attack due to both the lack of an adequate range of defensive enzymes resulting from the restricted distribution of cytoplasmic space and the susceptibility of sperm membrane lipids caused by significant amounts of polyunsaturated fatty acids. Under stressful conditions, spermatozoa undergo an intrinsic apoptotic pathway characterized by the generation of mitochondrial ROS, the loss of mitochondrial membrane potential, caspase activation, phosphatidylserine exposure, and oxidative DNA damage [15].

Antioxidant defense is essential for an adequate sperm–oocyte interaction. Under physiological conditions, ROS are converted by the action of the antioxidant enzyme SOD into hydrogen peroxide, which is metabolized by CAT and GPX into water and oxygen, thus lowering intracellular stress [16]. The imbalance of these antioxidant defense mechanisms is correlated with an impairment of ejaculate quality, with testicular morphological changes leading to male infertility [17].

The environment, lifestyle, and the resulting OS can also induce epigenetic variations, post-translational modifications of histones, coding and non-coding RNAs, and DNA methylation, which are closely linked to spermiogenesis alteration and male infertility [18]. A distinctive sign of spermiogenesis is the change in the structure of the chromatin, resulting from the exchange of the majority of histones with protamines, which occurs precisely following the information of the epigenetic memory, and this is important for the compaction of the nuclear chromatin, the maturation of the spermatozoa, and fertility [19]. During the histone-to-protamine transition, epigenetic regulators work together to facilitate the reorganization of the paternal genome in highly condensed sperm nuclei through histone modification. It has been hypothesized that the changes induced in the sperm epigenome are profound, physiological, dynamic, probably irreversible, and therefore lead to infertility [20]. OS and genetics can therefore compromise the quality of sperm and its ability to fertilize the oocyte.

It is known that OS disrupts DNA integrity through the aberrant recombination of repetitive sequences. If these mutations occur at the level of genes involved in spermatogenesis such as Y repetitive sequences, then an impact on reproductive health is achievable [21]. Similarly, translocations of chromosome 15 are correlated with infertility. Chromosome 15 includes several genes involved in spermatogenesis, such as cation channel sperm-associated protein (*CATSPER*) and the related sensorineural hearing loss (*STRC*). Translocations in these genetic portions due to OS are correlated with a lack of sperm motility, and therefore infertility, and are also associated with hearing loss [22,23,24].

Various methodologies have been developed to evaluate the levels of specific OS biomarkers in men with fertility complications.

A biomarker is the assessment of a biological, pathological, or pharmacological process. In the fertility context, a biomarker is a biological measurement that provides information about reproductive health or the ability to conceive.

**Figure 1:**
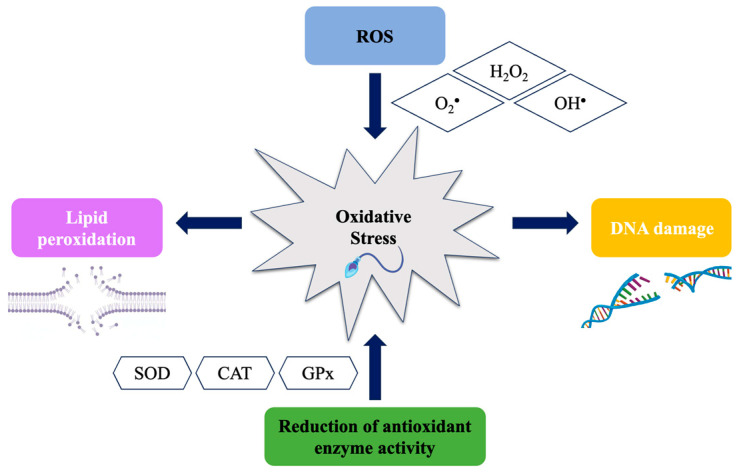
The main consequences of oxidative stress in sperm due to the formation of reactive oxygen species (ROS) and/or reduction of antioxidant enzyme activity.

OS biomarkers are molecules or enzymes indicating increased levels of OS and cellular damage associated with it [25]. Traditionally, OS biomarkers used in male infertility include DNA damage, lipid peroxidation, and antioxidant enzyme activity (Figure 1). However, these biomarkers are not always reliable and can be influenced by various factors, such as age, behavioral factors (e.g., nutrition and lifestyle), and concomitant pathologies. Therefore, in recent years, numerous research has been conducted to identify new OS biomarkers related to male infertility.

This evidence-based study aims to provide a comprehensive view on oxidative biomarkers in male infertility and new methodologies and techniques developed to evaluate OS in infertile men. A transcriptome analysis to identify genes regulated during OS, providing information on the metabolic pathways involved in the antioxidant response, will also be conducted. The application of these new methodologies has provided important information on the mechanisms underlying male infertility and has opened new perspectives for treatment. Additionally, evaluating biomarkers can help identify patients who may benefit from specific treatments.

## 2. Critical Steps of Spermatogenesis

Spermatogenesis is a complex process that leads to the formation of numerous mature haploid spermatozoa capable of crossing the female reproductive tract and fertilizing the oocyte. It is divided into three distinct phases: Initially, spermatogonia proliferate to give rise to primary spermatocytes, which then undergo two meiotic divisions resulting in the production of haploid spermatids. The final phase of the process, spermiogenesis, involves the maturation and final morphological transformation of spermatids into mature spermatozoa [26].

Spermatogenesis begins with spermatogonial stem cells, which through several mitotic divisions increase their population, leading to the formation of spermatogonia. Subsequently, these sperm cells undergo two successive meiotic divisions during which the chromosomes duplicate and undergo homologous recombination, resulting in the formation of genetically different tetraploid spermatocytes. A second meiotic division follows, leading to the production of round haploid spermatids. These round cells undergo spermiogenesis, during which remodeling of the spermatozoa occurs and they acquire an acrosome and a flagellum, essential for sperm motility and crossing the zona pellucida of the oocyte [27].

During spermatogenesis, the sperm undergoes molecular remodeling at the nuclear level essential to reduce cell volume and protect the genetic content [28]. Starting from this phase, DNA begins to be highly compact through the replacement of histones with protamines in the spermatid nucleus [29,30]. Over the process of protamination, histones are first hyperacetylated and then replaced with transition proteins (TP1 and TP2), which are finally removed and replaced with protamines [31].

In mammals, this process occurs in the seminiferous tubules of the testis, and as the germ cells pass through each stage, they are translocated from the base of the seminiferous epithelium to the luminal border. At the end of the spermatogenesis, spermiation occurs, through which mature haploid spermatozoa are released into the lumen of the seminiferous tube. The process is characterized by supporting cells, such as Sertoli cells, which provide structural and nutritional support during spermatogenesis and directly facilitate the translocation of germ cells within the epithelium. Furthermore, cytoskeletal elements are essential for motility, differentiation, migration, adhesion, and intracellular trafficking [27].

Although ROS, along with various factors in spermatozoa, increase the production of intracellular cAMP, which activates protein Kinase A (PKA), thereby inducing an increase in tyrosine phosphorylation, the main driving force behind sperm hypermotility and subsequent acrosome reaction [32], mature spermatozoa and the different stages of spermatogenesis are particularly susceptible to damage caused by ROS [33].

Since the process of chromatin condensation, involving the replacement of histones with protamines essential for correct sperm functionality, occurs in the spermatid nucleus, it is particularly susceptible to damage induced by ROS [30,33,34].

ROS can alter sperm DNA, causing DNA strand breaks, epigenetic mutations, and polymorphisms. If the damage is mild, then spermatozoa are able to repair it; however, extensive damage leads to DNA fragmentation and apoptosis, decreasing the quality of the seminal fluid [35]. Furthermore, the sperm membrane contains high levels of polyunsaturated fatty acids, which are the main targets of ROS, thereby reducing sperm motility [36].

## 3. Male Oxidative Stress Infertility (MOSI) Biomarkers

There is no doubt that standard semen analysis by verifying the morphology, number, and motility of the spermatozoa remains the point of reference for the diagnosis and treatment of male infertility; however, the awareness that OS affects normal fertility increasingly drives clinicians and researchers to investigate the oxidative state of infertile patients to establish targeted therapy. Agarwal et al. (2016) [37] proposed the term male oxidative stress infertility, or MOSI, as a new descriptor for infertile men with altered semen parameters, which also includes patients previously classified as having idiopathic male infertility.

It is estimated that infertility appears in 25–80% of infertile men with low levels of antioxidants in sperm. Considering that spermatozoa are exposed to both internally produced ROS and those present in the seminal plasma due to external factors such as smoking, alcohol, heat, and others, determining their levels is of fundamental importance as they can be a cause of reduced sperm quality with a decrease in motility and vitality and increased sperm DNA fragmentation [38,39,40].

The standard of excellence for OS biomarkers is 8-hydroxy-2′-deoxyguanosine (8-OHdG), a product of DNA peroxidation [41]. 8-OHdG can be measured in the plasma, semen, and urine and has been shown to be significantly higher in infertile men than in fertile men [42]. This indicates increased DNA damage and the deterioration of sperm function. Some studies have demonstrated a correlation between 8-OHdG levels and the severity of male infertility, suggesting that it could be used as a prognostic biomarker to predict the response to treatment. The standardization of probes to detect DNA oxidation in the form of 8-OHdG has been used as a biomarker of OS in a reproductive context [43].

**Figure 2:**
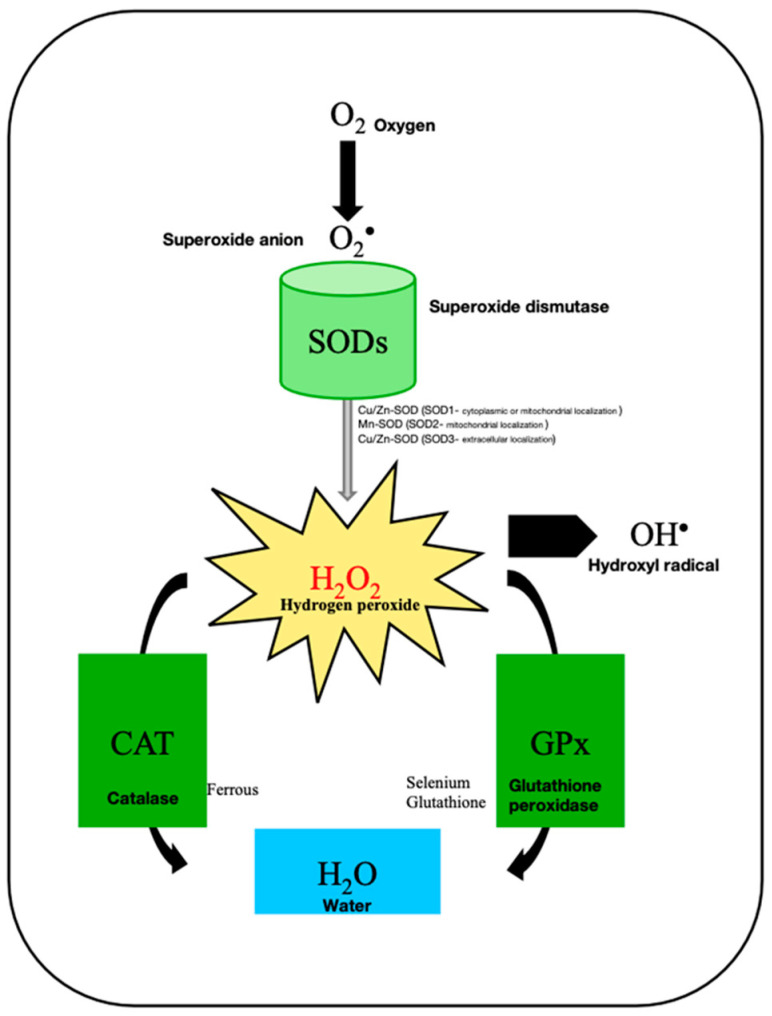
Generation of ROS from sequential transfer of electrons from complete reduction to water.

In addition to these biomarkers, various other molecules and enzymes have been identified to evaluate OS in male infertility. Alterations in the expression of antioxidant enzymes, such as superoxide dismutase (SOD), which catalyzes the cleavage of superoxide anion radicals into hydrogen peroxide, catalase (CAT), and glutathione peroxidase (GPx), which decreases the hydrogen peroxide content in cells through the formation of water and oxygen [44] (Figure 2), have been reported in infertile patients [45] and correlate with a reduced fertilizing capacity [46] due to DNA fragmentation and sperm abnormalities [47].

Furthermore, proteomic studies have shown that the expression of proteins associated with spermatogenesis and the regulation of oxidative stress (OS) is altered in infertile men with high levels of seminal reactive oxygen species (ROS).

Agarwal and colleagues (2019) [48] assessing the overexpression of 248 proteins in idiopathic infertile men found overexpressed proteins regulated by two transcription factors (TFs), peroxisome proliferator-activated receptor gamma coactivator 1-alpha (PPARGC1A) and nuclear factor erythroid 2-related factor 2 (NFE2L2), which are the co-activator and activator of antioxidant enzyme transcription, respectively. PPARGC1A is implicated in ROS detoxification and serves as a co-activator of transcription, involved in activating antioxidant defense systems [49]. Similarly, NFE2L2 acts as a transcriptional activator of cellular antioxidant enzymes [50].

According to Agarwal et al. (2019) [48], because transcription and translation are minimal in ejaculated spermatozoa, they hypothesized that the overexpression of certain proteins, regulated by the TFs PPARGC1A and NFE2L2, is due to increased activity of these transcription factors during spermatogenesis. Meanwhile, Fujii and Imai (2014) [51] described GPX4 as playing an essential role in spermatogenesis, and therefore male infertility, by identifying that mitochondrial GPX4 variants determine structural abnormalities in the central part of the sperm. Furthermore, the absence of GPX4 at the sperm nuclear level is correlated with impaired chromatin condensation, nuclear instability, and subsequent DNA damage [52]. Finally, mitochondrial peroxiredoxin-5 (PRDX5) is also involved in the antioxidant defense mechanism in sperm mitochondria [53]. Therefore, alterations in the expression of spermatogenesis-related proteins mediated by seminal ROS may be associated with infertility and screened as biomarkers of infertility associated with oxidative stress.

An innovative method to evaluate the levels of oxidation–reduction potential (ORP) in whole seminal fluid is the MiOXSYS (male infertility oxidative system) technology [37]. ORP is a useful clinical biomarker for the direct measurement of OS in biological samples. In a recent multicenter study on 2092 semen samples, ORP was negatively correlated with count, motility, and normal forms of spermatozoa [54]. Furthermore, a prospective observational study revealed that seminal ORP significantly influences reproductive outcomes in cases of artificial reproductive technology (ART), acting as a biomarker of good fertilization, blastocyst development, and implantation [55]. However, some authors have suggested that the elimination of seminal plasma may cause the increased sensitivity of spermatozoa to OS inducers, which could influence the outcome, resulting in misleading results.

Another promising biomarker involved in the regulation of sperm function is nitric oxide (NO) and its derivatives [41]. NO is an important chemical messenger that plays important functions in regulating junctions, controlling cytokine and hormone levels in the testes, as well as supporting the development of germ cells [56].

However, high concentrations of NO and heat-induced nitric oxide synthase (iNOS), an enzyme involved in the production of NO, have been shown to be increased in infertile patients compared to fertile ones and responsible for sperm DNA damage and decreased sperm motility [57,58].

Although low concentrations of NO are essential for motility and the capacitation process, high concentrations of NO can induce DNA damage in different ways [59]. In fact, it can create DNA strand breaks caused by the formation of NO-dependent reactive species such as peroxynitrite, which directly attack the DNA and cause failure to correct single-strand breaks, leading to the formation of double-strand breaks, which are probably responsible for cell death. Furthermore, NO can act indirectly on DNA by interacting with other cellular factors and biological processes that alter DNA, such as the activation of endonucleases, the induction of apoptosis, and the inhibition of DNA repair enzymes. The presence of NO can induce DNA deamination, such as the deamination of guanine to xanthine, which causes depurination to form basic sites in DNA, resulting in single-strand breaks or incorrect repairs [60].

Therefore, it has been hypothesized that measuring the levels of NO and its derivatives in plasma and seminal fluid may provide significant information on OS and sperm function. Some preliminary studies have shown increased levels of NO and its derivatives in infertile patients compared to fertile ones, suggesting that they may be useful in diagnosing male infertility [61,62,63].

Increased glucose and lipid levels can also be considered as markers of fertility. In fact, their high levels can cause an excessive supply of energy substrates to the metabolic pathways of adipose and non-adipose cells, which leads to a high production of ROS [64]. OS is directly related to the presence of the glucocorticoid, which causes an increase in the flow of electrons at the level of the electron transport chain, which by directly influencing the hypothalamic–gonadal axis can directly affect the reproduction and functionality of the sperm [65,66].

Another important factor related to fertility is the level of trace elements, in particular zinc, copper, and magnesium, which play a fundamental role in the process of male reproductive development [67]. In fact, the imbalance of these elements is correlated with the increase in OS, affecting the reduced motility of spermatozoa. Zn, which is known for its antioxidant properties, has an important role in sperm motility, as it is involved in the formation of the disulfide bridge during sperm maturation [68]. Similarly, low levels of Mg, probably due to the presence of chelating agents, can be the cause of premature ejaculation and infertility [67].

Malondialdehyde (MDA) is the main indicator of lipid peroxidation [69]. The presence of ROS causes peroxidation cycles, resulting in the fragmentation of sperm DNA and the oxidation of membrane lipids present in sperm. Elevated levels of MDA are closely related to a decrease in sperm motility, thus indicating the negative impact of OS on motility [70].

It follows that the evaluation of the oxidative state and antioxidant defenses assessed by a methodology easily reproducible in the laboratory can be considered an important tool for the diagnosis of male infertility.

## 4. Established Methods to Detect OS Biomarkers

Numerous analytical techniques, in recent years, have been used to develop methods for quantifying oxidative stress.

In past years, 8-OHdG was examined by HPLC coupled with electrochemical detection, but this method was abandoned as it was strongly influenced by the possible spontaneous formation of this molecule during the sperm DNA extraction phase [71]. Currently, 8-OHdG in sperm is measured by direct immunofluorescence with the use of specific antibodies labeled with FITC fluorochrome. The result is then quantified by flow cytometry [72]. However, fluorescence can also be monitored by fluorescence microscopy. Despite the high precision of modern flow cytometry equipment and the sensitivity of fluorescence techniques, the costs of such instruments can be limiting. Additionally, the experience and skills of the operator are crucial for correctly interpreting the results.

The activity of antioxidant enzymes, such as CAT, SOD, and GSH, are measured by colorimetric tests, using specific reagents and a spectrophotometer on plasma samples [73]. Spectrophotometers measure absorbance at different light wavelengths, enabling the determination of substance quantity in a solution. Due to its affordability, speed, and lack of reliance on specialized equipment, this procedure can be conducted in any clinical or research laboratory.

The methodological breakthrough in protein detection has been achieved through the combination of mass spectrometry with liquid chromatography (LC–MS/MS) [48]. This technique is widely used in biochemistry, molecular biology, and pharmacology to characterize proteins, metabolites, and other chemical compounds [74]. In short, the chromatograph separates the compounds present in the sample, while two or more mass spectrometers in series act as detectors. Compounds are selected and fragmented within the first mass analyzer, and the resulting fragments are further analyzed in the second mass analyzer [75].

This technique offers extremely high sensitivity and specificity, allowing for more detailed information on the structure and chemical composition of the molecules under study. This leads to a significant reduction in diagnostic errors.

The innovative method MiOXSYS involves the potential for electrons to move from one species to another. To reduce the oxidative effect of toxins and prevent the ability of oxidants to acquire electrons from other structures and cause DNA damage, antioxidants donate electrons to oxidant species. Therefore, measuring ORP levels, and the relationship between oxidants and antioxidants, allows for a comprehensive measurement of OS [76]. Subsequently, an ORP cutoff value of 1.34 mV/106 sperm/mL has also been recognized, beyond which reproductive capabilities are compromised [77]. Measurements are performed on completely liquefied seminal samples [78]. The test, performed on seminal fluid within 30 min of emission, involves applying a small portion of semen to a sensor inserted into the analyzer, and is capable of providing a result after just 2 min. Unlike other markers of OS, this method allows for the direct evaluation of the ORP of seminal fluid, presenting numerous advantages, including a short duration, simplicity of execution, and the small volume of sample required [79].

Glucose and lipid levels are measured in blood samples, using specific kits. In particular, the enzyme glucose oxidase allows us to evaluate fasting plasma glucose; instead, lipids are measured as total cholesterol (TC) and serum triglyceride (TG). Through an enzymatic colorimetric method, TC is measured using two enzymes: cholesterol esterase and cholesterol oxidase. Finally, TGs are assayed using glycerol phosphate [80].

The analysis of trace elements in seminal fluid is obtained using the atomic absorption spectrophotometer (AAS) with the use of high-purity reagents [81]. AAS utilizes the interaction between light and matter to quantitatively determine the presence of a metal in a solution. Based on the principle that each element in the periodic table has a specific structure of orbitals and electrons, this technique allows for the identification and quantification of the number of atoms of an element in the solution by measuring the amount of light absorbed at precise wavelengths, which is characteristic of the orbital configuration of that element. Also in this case, limits are related to the equipment.

Lastly, malondialdehyde (MDA) is quantified by a colorimetric method, using the reaction of thiobarbituric acid, which allows for the detection of TBARS, i.e., thiobarbituric acid reactive substances. These substances are formed during lipid peroxidation and are highlighted by a pink product following the reaction. The color intensity at 535 nm is directly proportional to the concentration of TBARS in the sample. Since TBARS are mainly represented by MDA, they provide a measure of MDA in semen [82,83].

## 5. New Oxidative Biomarkers in Male Infertility

### 5.1. NGS Polymorphisms

Many researchers agree on the need to conduct innovative investigations that can provide more data for the evaluation of the real reproductive health state, increasingly using emerging technologies. OS dosage represents a very valid infertility screening approach, especially in cases of idiopathic male infertility. Furthermore, advances in molecular biology and bioinformatics now highlight alterations in genes involved in redox mechanisms in infertile males.

It is known that OS is correlated with alterations in genetic material. Several studies demonstrate that free radicals induce changes in the genetic material, that is, polymorphic variations [84]. In 50% of cases of idiopathic non-obstructive azoospermia (INOA), it has an unidentified genetic basis, and this suggests that the polymorphism of genes in autosomal chromosomes may also play an important role in spermatogenesis [85]. In the study conducted by Cannarella and co-workers (2020) [25], 15 genes involved in the process of spermatogenesis were analyzed in patients with idiopathic oligozoospermia or NOA. Among the different genes analyzed, we find those that induce meiotic arrest (testis expressed gene 15 meiosis and synapsis associated (*TEX15*), synaptonemal complex central element protein 1 (*SYCE1*)*,* testis expressed 11 (*TEX11*)) or maturation arrest at the spermatocyte stage (nuclear receptor subfamily 5 group A member 1 (*NR5A1*), synaptonemal complex protein 3 (*SYCP3*), zinc finger MYND-type containing 15 (*ZMYND15*), meiosis specific with OB-fold (*MEIOB*), and heat shock transcription factor 2 (*HSF2*)). For example, *NR5A1* regulates the expression of steroidogenic genes such as 3 beta- and steroid delta-isomerase (*3β-HSD*) in Leydig cells.

The impact of external factors such as metal nanoparticles, which induce OS, on the expression of genes associated with steroidogenesis have been evaluated [86,87]. The analysis by Hussein and colleagues (2016) [88] determined that exposure to zinc oxide nanoparticles reduced the expression of nuclear receptor subfamily 5 group A member 1 (*NR5A1*)*,* hydroxysteroid 17-beta dehydrogenase (*17β-HSD*), 3 beta- and steroid delta-isomerase *(3β-HSD)*, and testosterone, thus compromising male fertility.

Heat shock factors (HSFs) are transcriptional regulators of heat shock protein (HSP), involved in cellular protection against proteotoxic damage [89]. In particular, heat shock transcription factor 1 (*HSF1*) and heat shock transcription factor 2 (*HSF2*) are involved in cell differentiation and spermatogenesis. The constitutively active expression of *HSF1* has been assessed to block spermatogenesis and induce programmed cell death. In contrast, low levels of *HSF2* expression result in increased apoptosis and reduced sperm count. Therefore, *HSF1* and *HSF2* could represent a marker to identify the accumulation of damaged proteins and the induction of cell death [90].

Some novel targets of male infertility were reported by Cannarella and co-workers (2020) [25]. They provided updated insights into the molecular biology of spermatogenesis, underlining the role of the molecular factors involved in oocyte fertilization and embryo growth that are produced during spermatogenesis and carried out by the sperm cell. The authors highlighted the overall contribution of the sperm genome (including epigenetic regulation), transcriptome, and proteome to embryo formation and development, which needs to be investigated to identify novel molecular targets responsible for male infertility.

Currently, among the most used techniques in the study of genetic polymorphisms of interest, next-generation sequencing (NGS) occupies an important place. This term refers to the set of nucleic-acid-sequencing technologies that possess the ability to sequence millions of DNA fragments in parallel [91]. For these reasons, NGS technologies have changed the way we approach genome analysis. The analysis of some gene polymorphisms can quickly provide more in-depth answers, even for unexplained infertility [92]. In fact, the NGS technique allows for the simultaneous analysis of a panel of genes for the study of the main causes of male infertility including azoospermia, asthenozoospermia, and defects in sperm morphology [93].

Polymorphic variations, induced by OS, can lead to oligozoospermia or azoospermia and can be identified through NGS.

In the study conducted by Salvi and co-workers (2022) [94], data on the Genome Wide Associations Studies (GWAS) platform were used to identify the presence of polymorphisms associated with infertility. The results found polymorphic variants, associated with infertility, in the major histocompatibility complex, class II, DR alpha (*HLA-DRA*) gene: rs8084 and rs7192, which control spermatogenesis in men and are involved in cell adhesion and motility at the level of spermatocytes and spermatids. Another polymorphism in the same gene is rs7194, which is associated with non-obstructive azoospermia [95].

Several studies have found a cause of idiopathic male infertility in the strong correlation between OS levels, due to high fluoride levels, and the expression of glutathione S-transferase (*GST*). *GST* is a gene family of isozymes that catalyze the conversion of hydrogen peroxide to water [96]. A target of OS at the sperm level is the mitochondrion, which reduces the source of ATP and therefore sperm motility [97].

The study conducted by He and co-workers (2023) [98] demonstrated that genetic variations in human *GTS*, in particular in the glutathione S-transferase mu 1 (*GSTM1*), glutathione S-transferase theta 1 (*GSTT1*), and glutathione S-transferase pi 1 (*GSTP1*) genes, are correlated with sperm motility and concentration. The authors highlighted that the present *GSTM1* and *GSTT1* genotypes, as well as the wild-type *GSTP1* genotype, are associated with high levels of 8-OHdG and MDA and low levels of antioxidant activity.

Therefore, we can say that the search, through NGS, for genetic variants could represent a good biomarker of idiopathic male infertility.

Furthermore, the application of this technique to the transcriptome has focused attention on the potential functional role of new types of non-coding RNAs, which could become innovative diagnostic markers and/or therapeutic targets in the near future. The formation of a mature spermatozoon requires the expression of a number of coding and non-coding genes. The altered expression of exosomes including tRNA, piwi-RNA, and ribosomal RNA can lead to arrested spermatogenesis [99].

The expression of genes such as protamine 2 (*PRM2*) and deleted in azoospermia (*DAZ*), which can be affected by lifestyle and used as molecular markers in NOA patients, has been evaluated to predict the absence of mature spermatozoa [100]. Indeed, the low-protein diet in a study conducted on male mice was associated with changes in small RNA profiles in epididymosomes and mature spermatozoa, suggesting the interaction and transfer of extracellular vesicles to the sperm during epididymal transit [101].

Therefore, although the quantity of sperm RNA is negligible, it is important for investigating the causes of male infertility. New results from microarray, NGS, and RNAseq techniques led to the discovery of new transcripts in sperm, useful as clinical markers of male infertility [102].

### 5.2. Differentially Expressed Genes (DEGs)

Another type of potential biomarker of male infertility is described in an interesting recent study aimed to identify some DEGs involved in non-obstructive azoospermia (NOA) and overall male infertility [103,104] through transcriptome sequencing to detect differences in mRNA expression in testicular tissue [105]. The authors demonstrated that of the 25 DEGs investigated, 8 genes (testicular haploid expressed gene (*THEG*), spermatogenesis associated 20 (*SPATA20*), rhophilin associated tail protein 1 like (*ROPN1L*), glutathione S-transferase F1 (*GSTF1*), serine kinase 1B (*TSSK1B*), calcium binding protein, spermatid associated 1 (*CABS1*), adenosine deaminase domain containing 1 (*ADAD1*), and RIMS binding protein 3 (*RIMBP3*)) are involved in the spermatogenic process or in specific phases of spermatogenesis, and hypothesized that the alteration in the expression of these genes leads to impaired spermatogenesis and, therefore, to male infertility.

In particular, the serine kinase 1B (*TSSK1B*) gene participates in spermatogenesis and it is responsible for proper morphogenesis and differentiation after meiosis when spermatid elongation occurs [106].

Similarly, *SPATA* genes have been identified as testis-specific genes that regulate apoptosis during zebrafish spermatogenesis [107]. Zheng and co-workers (2017) [108] evaluated its role in the sperm DNA hydroxymethylation of men exposed to bisphenol A (BPA). BPA exposure negatively affected *SPATA20* gene expression. Thus, it is suggested that *SPATA20* regulates the response following sperm DNA damage, thus influencing ejaculate quality. *SPATA20* is a thioredoxin-like protein, which catalyzes sulfide bonds and regulates the expression of antioxidant enzymes [109]. Therefore, *SPATA20* can probably have a protective role towards the formation of ROS and represents a good marker of OS.

Sertoli cell-only syndrome (SCOS) is the most severe pathological type of non-obstructive azoospermia. Present studies have analyzed, through RNA sequencing, the DEGs in patients with SCOS. The authors highlighted that the expression of three DEG genes (caspase 4 (*CASP4*), caspase 1 (*CASP1*), and phospholipase A2 group IVA (*PLA2G4A*)) were upregulated in SCOS patients. *CASP4* and *CASP1* are part of the caspase family and participate in cellular pyroptosis. It is known that there is a direct correlation between inflammation and OS with cellular pyroptosis as cellular pyroptosis is an inflammatory form of cell death [110]. CASP4 and CASP1 are pro-apoptotic proteins, which in the presence of ROS and the consequent OS, induce mitophagy and apoptosis [111]. Testicular inflammation, and the subsequent OS, induces changes in spermatogenesis and sperm transport and function [112].

In view of all this, the present studies therefore conclude that these genes could be used as potential biomarkers for the early detection of NOA.

## 6. Epigenetic Mechanisms during Spermatogenesis and Epigenetic Factors in OS Affecting Male Infertility

The occurrence of new technologies has revealed differential expression patterns of various genes in both immature male germ cells and sperm, which have opened a new space into the etiology of male infertility.

More and more evidence has demonstrated that epigenetic modifications are important factors regulating spermatogenesis in all its phases [113]. These alterations also control the pattern of expression in various genes during spermatogenesis and fertilization under regular biological conditions [114]. Recent studies reveal that several genes in testicular cells are regulated through the epigenetic process, which indicates the critical role of the epigenetic machinery in spermatogenesis, sperm development or maturation, the fertilization process, and male fertility [25].

**Figure 3:**
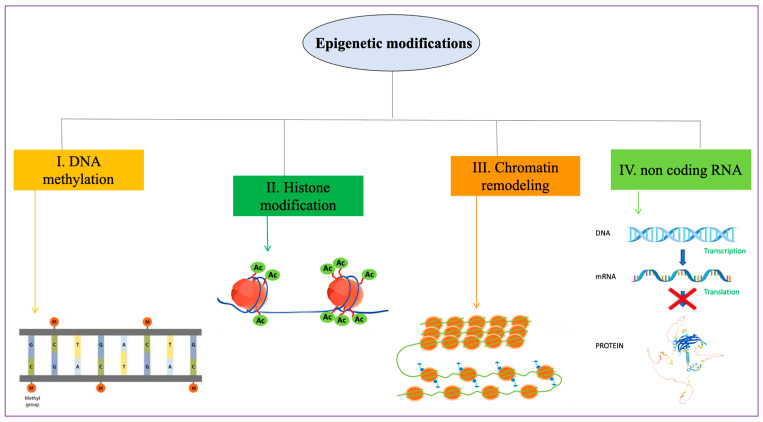
Graphical illustration of epigenetic modifications. These can be divided into 4 classes: (I) DNA methylation, (II) histone modifications, (III) chromatin remodeling, and (IV) non-coding RNA regulation.

Epigenetics represents the process of DNA modification influencing cell differentiation [115]. The definition of epigenetics refers to molecular changes in DNA that can regulate genetic activity without changing the DNA sequence [116,117]. These modifications include DNA methylation, post-translational histone modifications, chromatin rearrangement, and non-coding RNAs (ncRNAs) [118] (Figure 3).

DNA methylation consists of the binding of a methyl group to carbon 5 of a cytosine next to a guanine (CpG) by the enzyme DNA methyltransferase (DNMT) and generally involves the inactivation of gene expression [119]. Sperm DNA hypomethylation of the H19 gene and hypermethylation of the mesoderm-specific transcript (MEST) and small nuclear ribonucleoprotein polypeptide N (SRNPN) genes have been associated with male infertility [120]. Moreover, alterations in DNA methylation at specific genomic loci have been associated with difficulty conceiving, and DNA methylation and gene transcription patterns may be predictive of assisted reproductive technology (ART) success rates [121,122,123]. Patients with a history of oligoasthenoteratozoospermia (OAT) have also been shown to have higher rates of DNA methylation and altered gene expression in embryos that underwent preimplantation genetic screening after in vitro fertilization (IVF) [124].

Histones are basic proteins which, by binding DNA, form the main components of chromatin. Modifications of their N-terminal tails, such as phosphorylation, methylation, acetylation, and ubiquitination, determine a relaxed or compact structure of the chromatin, which favors gene expression or not [125,126,127]. If this crucial process does not occur correctly due to external stimuli, then reproductive capabilities can be severely compromised. Sperm cells with reduced levels of protamine content have been shown to have increased susceptibility to DNA damage and reduced viability [128].

MicroRNAs (miRNAs) refer to single-stranded RNAs about 20 nucleotides long involved in the regulation of gene expression at the transcriptional and post-transcriptional levels [129]. They inhibit gene expression because they interact directly with messenger RNA molecules [116], preventing their translation or inducing their degradation [130]. In the last years, several studies have reported new information on the role of a class of non-coding RNAs on the production and fertilizing potential of sperm [131].

An increasing amount of evidence has shown that the dysregulation of various specific miRNAs can cause impaired spermatogenesis and male infertility [130]. A few studies show that the profiles of extracellular miRNAs are related to environmental factors and lifestyle [132,133,134].

In the study conducted by Ferrero and co-workers (2024) [135], the expression of the miRNA and the exposure to environmental contaminants was correlated. Their results highlighted a reduction in the expression of some miRNAs (including microRNA let-7c (let-7c-5p), microRNA let-7f-1 (let-7f-5p), microRNA 23a (miR-23a-3p), microRNA 23b (miR-23b-3p), and microRNA 320a (miR-320a-3p)), which are strictly regulated during spermatogenesis. miR-23b-3p and miR-320a-3p can alter the expression of many specific genes in spermatogenesis such as 6-phosphofructo-2-kinase/fructose-2,6-biphosphatase 4 (PFKFB4), hyaluronan mediated motility receptor (HMMR), spermatogenesis associated 6 (SPATA6), testis expressed 15, meiosis and synapsis associated (TEX15), SRY-box transcription factor 6 (SOX6), and nucleolar protein 4 (NOL4). Being involved in the spermatogenesis process, their altered expression due to an increased aberrant expression of miRNA could compromise male fertility [136].

Long non-coding RNAs (lncRNAs), sequences longer than 200 bp, also play a key role in the regulation of various cellular processes. lncRNAs regulate the expression of coding genes through epigenetic modifiers such as DNA methylation and histone modifications.

The expression of lncRNAs may serve as a biomarker of spermatogenesis regulation [137]. Zhang et al. (2015) [138] correlated sperm HOTAIR (Hox transcript antisense intergenic RNA) levels, which is involved in tumor development and progression, with sperm Nrf2 (nuclear factor erythroid 2–related factor 2) expression levels. Low levels of Nrf2 expression are associated with semen quality and a reduction in antioxidant gene expression [139], particularly in asthenozoospermic and oligoasthenozoospermic patients. This association may be attributed to low levels of histone H4 acetylation at the Nrf2 gene promoter, leading to Nrf2 transcriptional inactivation [139,140]. According to this study, Nrf2 expression is regulated by HOTAIR through the hyperacetylation of histone H4 in the Nrf2 promoter, indicating a correlation between Nrf2 and HOTAIR expression levels in spermatocytes. Furthermore, superoxide dismutase (SOD) activity is directly proportional to reduced HOTAIR expression, thereby increasing OS and compromising sperm viability and motility.

This supports the evidence that epigenetic phenomena respond to environmental influences with a much greater plasticity than that inherent in the DNA sequence [141]. In fact, the most interesting and important hypothesis arising from studies on the sperm epigenome is the potential correlation between environmental influences and impaired fertility [142].

As predicted, ROS can induce epigenetic changes in sperm DNA, altering DNA methylation, histone modifications, and miRNA regulation [143,144,145].

Studies in mice have shown that male germ cells deficient in DNA methyltransferase 3-like (Dnmt3L) activity, crucial for proper DNA methylation, exhibit incomplete gametogenesis. Similarly, in humans, global hypomethylation mediated by ROS has been linked to Sertoli cell-only syndrome, testicular tumors, and hypospermatogenesis [143,146], just as some oxidizing agents are able to alkylate the protamines present in spermatozoa, compromising the condensation of spermatic chromatin [145].

Because the epigenome is influenced by the environment, numerous factors contribute to grow the reproductive pathology, including age, diet, drugs, and exposure to harmful substances [147,148]. Therefore, there is growing interest in determining which other epigenetic modifications might play a key regulatory role.

The use of epigenetic changes in DNA as markers to identify male factor infertility is challenging, as these changes can have little or marginal biological impact and, sometimes, different changes can lead to similar infertility phenotypes [149]. While determining causal relationships between these changes and embryological outcomes is objectively difficult, many sperm epigenetic changes have been associated with sperm abnormalities [115]. DNA methylation as a marker of male infertility is an attractive target, as it remains stable during spermatogenesis, unlike RNA transcription [150].

Unfortunately, it still remains to be clarified whether epigenetic changes in the sperm cell cause infertility and/or subfertility or whether the infertility is secondary to another disease [151].

The clinical implementation of epigenetic testing is promising, but no sufficient validation studies have yet been reported. However, the epigenetic profiling of sperm cells from infertile men is likely to be useful in the near future, both to assess the potential of sperm cells to contribute to normal embryogenesis and the risks associated with environmental exposures. We hope that other numerous studies on sperm epigenetic profiling can help understand the etiologic basis of compromised spermatogenesis [152].

## 7. Transgenerational Consequences of Epigenetic Changes in Sperm DNA

Modifications in sperm cells, such as DNA methylation and protamination, are thought to affect transcription during embryogenesis [150], and several studies suggest that these changes could also cause miscarriages [124].

In fact, environmental and lifestyle factors not only directly influence individuals, but also have transgenerational consequences, with parents potentially passing these effects on to their offspring [153].

Patients with a history of OAT have been shown to have altered gene expression in embryos that underwent preimplantation genetic screening after in vitro fertilization (IVF) [124].

As a matter of fact, recent studies show that parental exposure to environmental factors can influence fetal development and the health of offspring. Indeed, a recent study analyzing the consequences of male exposure to a specific environment before conception suggest that gene modifications induced by environmental toxins and lifestyle transmitted paternally can modulate offspring development through epigenetic inheritance [154].

Alterations in sperm DNA, caused by environmental exposure, are linked to both recurrent pregnancy losses and adverse health outcomes in children, including behavioral and physical disorders [155,156]. As well as exposure to contaminants, paternal use of alcohol and drugs (as cannabis and cocaine) can also lead to behavioral and cognitive alterations in offspring. In fact, excessive alcohol consumption alters the levels of DNA methylation of paternally imprinted and neurotrophic factor genes, in particular increasing paternally expressed gene 3 (*PEG3*). These variations are maintained in the cerebral cortex and in the ventral tegmental area in the offspring brain [157].

Additionally, the prolonged use of cocaine induces histone modifications, particularly of histone 3, which alter the expression of genes related to glutamate, stress, neurogenesis, and neurotrophic factor. These epigenetic changes are accompanied by memory deficits in both parents and offspring [113].

Data from studies on rodents have shown that preconcpeptional paternal exposure to delta-9-tetrahydrocannabinol (Δ9-THC), the most active cannabinoid, leads to deficits in cholinergic synaptic function in offspring, along with abnormalities in locomotor activity, impaired cognitive function, and long-lasting neurobehavioral effects. Additionally, exposure to Δ9-THC during adolescence in rats has been found to cause intergenerational effects on DNA methylation status in the nucleus accumbens (NAc) [158].

From a comprehensive literature review, it emerges that paternal exposure to BPA causes changes in sperm count and motility in both the father and offspring, as well as transgenerational heart and behavioral disorders. These findings have led to considering sperm as carriers of epigenetic memory, suggesting that this message, whether a simple phenotypic variation or a disease, can be transmitted across generations [159].

To conclude, an analysis conducted by Pabarja and co-workers (2021) [160] evaluated the synergistic effect of nicotine and alcohol on 25 adult mice and F1, which not only altered the quality of seminal fluid but also caused epigenetic modifications. Concurrently, their direct effect on the increase in ROS production was also evaluated. Indeed, serum MDA levels, as end products of lipid peroxidation and markers of OS, were significantly increased in all offspring.

Therefore, investigating sperm epigenetic alterations not only allows us to determine the cause of reduced male fertility but also to define its effect on future generations.

## 8. Insights into Future Redox Biomarker Research: Integrated Analysis of Omics Data for Male Infertility

Omics studies encompass various areas of research: genomics (focusing on genes), transcriptomics (examining transcription products), proteomics (analyzing proteins), and metabolomics (studying metabolites). [161]. Each one is complex and employs techniques that use large data sets that require technical expertise to analyze and highlight significant biological insights. Indeed, one of the major needs for a successful study in these areas is the collaboration of molecular biologists, geneticists, statisticians, and computational biologists. There are numerous databases available. Among these are the web servers “DiseaseConnect” (http://disease-connect.org, accessed on 23 January 2024), “RD-Connect Genome-Phenome Analysis Platform” (https://platform.rd-connect.eu/, accessed on 23 January 2024), and “Omics Database Generator” (https://github.com/jguhlin/odg, accessed on 23 January 2024).

A true multi-omics approach to studying spermatogenesis requires integrating data from each of these fields to gain deeper insight into the mechanisms that cause infertility [162]. Progress depends on improving the accessibility and functionality of public and interactive databases, as well as training researchers specialized in this type of data analysis. The databases use large-scale omics data to identify common genes and pathways to help identify related pathologies. This type of integrative analysis is important for the further understanding of the overall pathology, risks, and treatments of infertile males [163].

The omics approach holds promise for identifying unknown genetic factors and diagnosing male fertility issues in the near future. [164]. Among them, metabolomics is expected to become a very powerful tool for diagnostic testing [165]. Studies by Minai-Tehrani and colleagues in 2016 linked altered levels of oxidative stress (OS), glycerylphosphorylcholine, citrate, and lactate to male infertility [166]. Additionally, disciplines like glycomics and lipidomics have been shown to identify other diagnostic targets, such as elevated levels of arachidonic acid and other fatty acids in sperm [167]. Moreover, there is a suggestion that the seminal plasma glycome profile may correlate with male reproductive potential [168].

## 9. Conclusions and Future Perspective

The use of OS biomarkers may be advantageous for the diagnosis and treatment of male infertility. Accurate measurements of OS levels can provide valuable information about the severity of infertility and response to treatment. Based on the above, methods for assessing oxidative stress can be categorized as the direct analysis of reactive oxygen species (ROS) and methods capable of detecting ROS-induced alterations in proteins, lipids, and DNA. The current evidence shows that, in the absence of expensive tools for the detection of DEGs, proteins, or gene polymorphisms, the most immediate, simple, and cost-effective evaluation of oxidative status in seminal plasma is provided by the innovative MiOXSYS method, representing a system with high predictive value for male infertility. The ORP is an advanced measure of sperm function for oxidative stress, reliably predicting anomalies in elevated OS. Despite some limitations needing clarification, abnormal ORP levels can help identify altered sperm function, particularly in cases of idiopathic infertility. Ongoing studies aim to better understand the clinical significance of this new, reproducible measure of OS in assisted reproductive technology. In addition to assessing standard seminal fluid parameters, this method evaluates both ongoing damage and the effectiveness of antioxidant therapy. Consequently, it enables monitoring of effects, the adjustment of dosage or molecules, i.e., antioxidants in the short term, thereby preventing damage and enhancing patient management. Despite studies on the effects of antioxidant therapies still being controversial and further randomized controlled trials being necessary [169], some evidence from in vitro studies and animal models [170,171] provides interesting data on their efficacy and the type of formulation to be used.

However, further research is needed to evaluate the reliability and effectiveness of all the mentioned biomarkers. Above all, it is necessary to develop standardized and comparable measurement methods to ensure accurate and reproducible results so as to associate oxidation–reduction potential with multiple biomarkers in clinical practice for greater understanding and management of infertility.

Furthermore, longitudinal studies are needed to evaluate the effect of treatment on the trend of OS biomarkers over time.

Only through further research in this field will it be possible to improve the diagnosis and treatment of male infertility and provide hope to patients affected by this condition.

The constant and growing attention to omics technologies in the field of male reproductive health and the effects that OS produces on spermatozoa are supported by the increase in studies and scientific publications on the subject. Among them are those aimed at identifying biomarkers for the diagnosis of infertility and treatment options. However, these are relatively new fields of investigation, and the challenges are not lacking. While techniques, protocols, and analysis systems for genomics are already well established, for other omics there is still a long way to go. For example, it is already possible to sequence the genome of a single cell, but it is still not possible to analyze the proteome or metabolome at this level. The move to an integrative study of infertility on multiple omics levels would contribute to the understanding of the underlying pathological mechanism and would allow for the development of new diagnostic and therapeutic options.

## Data Availability

Not applicable.
